# The global pipeline of new medicines for the control and elimination of malaria

**DOI:** 10.1186/1475-2875-11-316

**Published:** 2012-09-07

**Authors:** Melinda P Anthony, Jeremy N Burrows, Stephan Duparc, Joerg JMoehrle, Timothy NC Wells

**Affiliations:** 1 Medicines for Malaria Venture (MMV), 20 rte de Pré-Bois 1215, Geneva, Switzerland

**Keywords:** Drugs, Resistance, Combinations, ACT, Endoperoxide, Spiroindolone, Relapse, Transmission

## Abstract

Over the past decade, there has been a transformation in the portfolio of medicines to combat malaria. New fixed-dose artemisinin combination therapy is available, with four different types having received approval from Stringent Regulatory Authorities or the World Health Organization (WHO). However, there is still scope for improvement. The Malaria Eradication Research agenda identified several gaps in the current portfolio. Simpler regimens, such as a single-dose cure are needed, compared with the current three-day treatment. In addition, new medicines that prevent transmission and also relapse are needed, but with better safety profiles than current medicines. There is also a big opportunity for new medicines to prevent reinfection and to provide chemoprotection. This study reviews the global portfolio of new medicines in development against malaria, as of the summer of 2012. Cell-based phenotypic screening, and ‘fast followers’ of clinically validated classes, mean that there are now many new classes of molecules starting in clinical development, especially for the blood stages of malaria. There remain significant gaps for medicines blocking transmission, preventing relapse, and long-duration molecules for chemoprotection. The nascent pipeline of new medicines is significantly stronger than five years ago. However, there are still risks ahead in clinical development and sustainable funding of clinical studies is vital if this early promise is going to be delivered.

## Background

Recent years have seen a transformation in the landscape of malaria drug discovery and development. A review of publicly available data from PubMed, clinicaltrials.gov as well as proprietary databases, such as Thomson Pharma, revealed that as of March 2012, over 50 projects were in progress worldwide. These include a wide variety of molecules in the window between late discovery (within 12 months of starting regulatory preclinical studies) and launch. Twenty-seven of these are in formal regulatory studies and beyond. In addition, in the past three years, five new medicines have either been approved by Stringent Regulatory Authorities [those that adhere to the guidelines of the International Commission for Harmonisation (ICH), or prequalified by the World Health Organization (WHO)].

The portfolio of new medicines contains several generations of products. The oldest group reflects the requirement for fixed-dose, artemisinin-combination therapy (ACT), driven by the need to protect artemisinin from the emergence and spread of resistance. This therapy is based around active molecules that were first identified over 30 years ago. The central focus for the development process of this therapy has been ensuring that the historical data available match current international standards of safety and efficacy.

The call for the eradication of malaria in 2007 led to a new research agenda [1] laying out priorities with broader horizons. As well as the focus on case management of uncomplicated and severe malaria, there is now a need for medicines that prevent transmission, stop relapse and provide chemoprotection against new infections. These characteristics are described by Target Product Profiles (TPPs), which give a description of the ideal medicine and the minimum acceptable profile. TPPs provide drug discoverers with a common standard of the unmet clinical needs in malaria control and eradication, thereby allowing a better focus. TPPs were developed with input from countries, field-based clinicians, and discovery and development teams [2], and are refined every two years [3].

## Methodology

This review aims to be as complete as possible, and not simply to be a review of projects supported by Medicines for Malaria Venture (MMV). To obtain such a global view, the data were obtained from publicly available sources, such as Medline, clinicaltrials.gov, and company websites, supported by commercial databases, such as Thomson Pharma ( http://www.thomson-pharma.com). There is unfortunately insufficient space to cite every single source. The data were updated in March-April 2012, although it is appreciated that the picture is continually evolving.

### Controlling malaria: artemisinin combination therapy as first-line treatment

**Figure 1:**
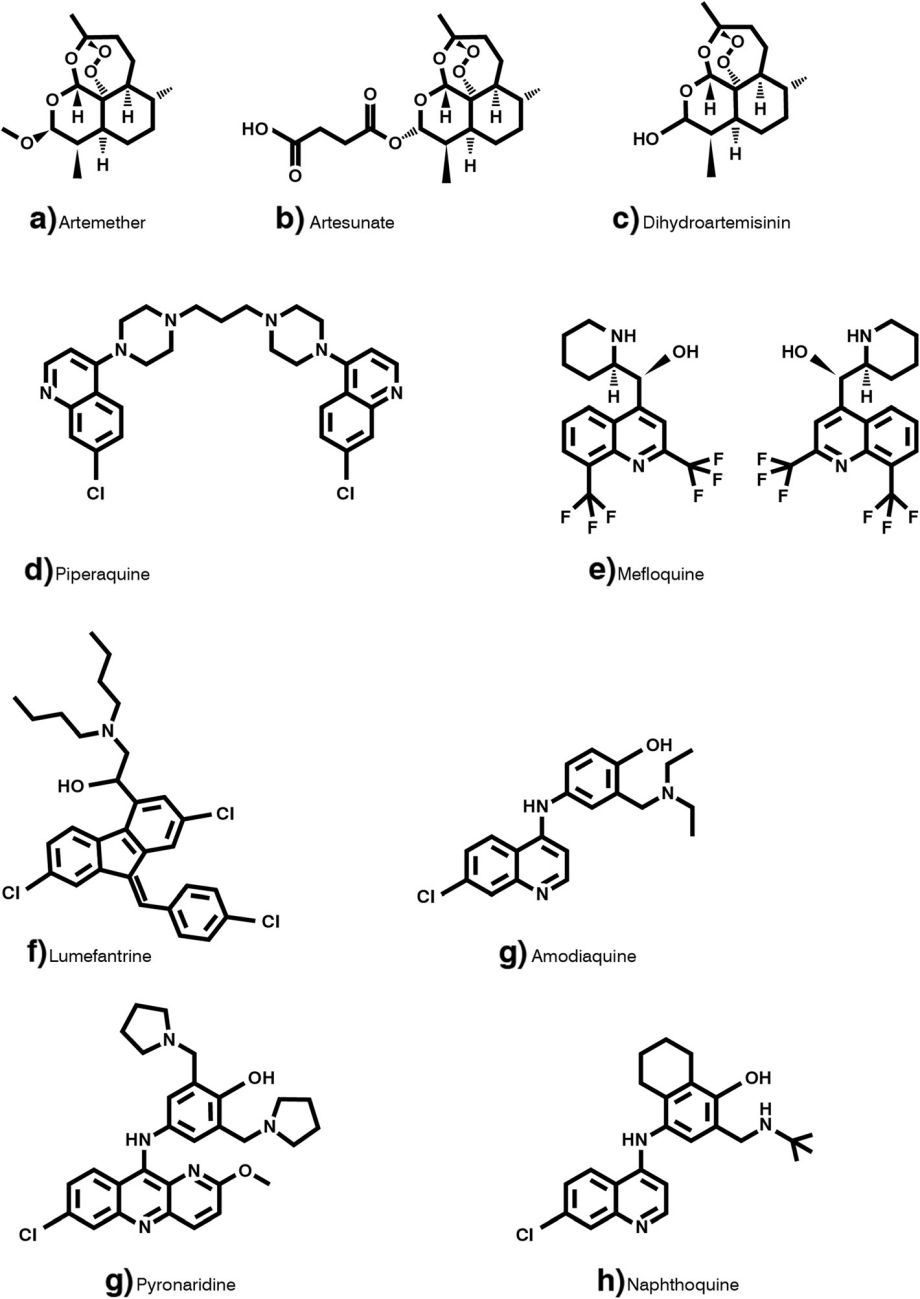
**Chemical structures of anti**-**malarials described in Table**1.

**Figure 2:**
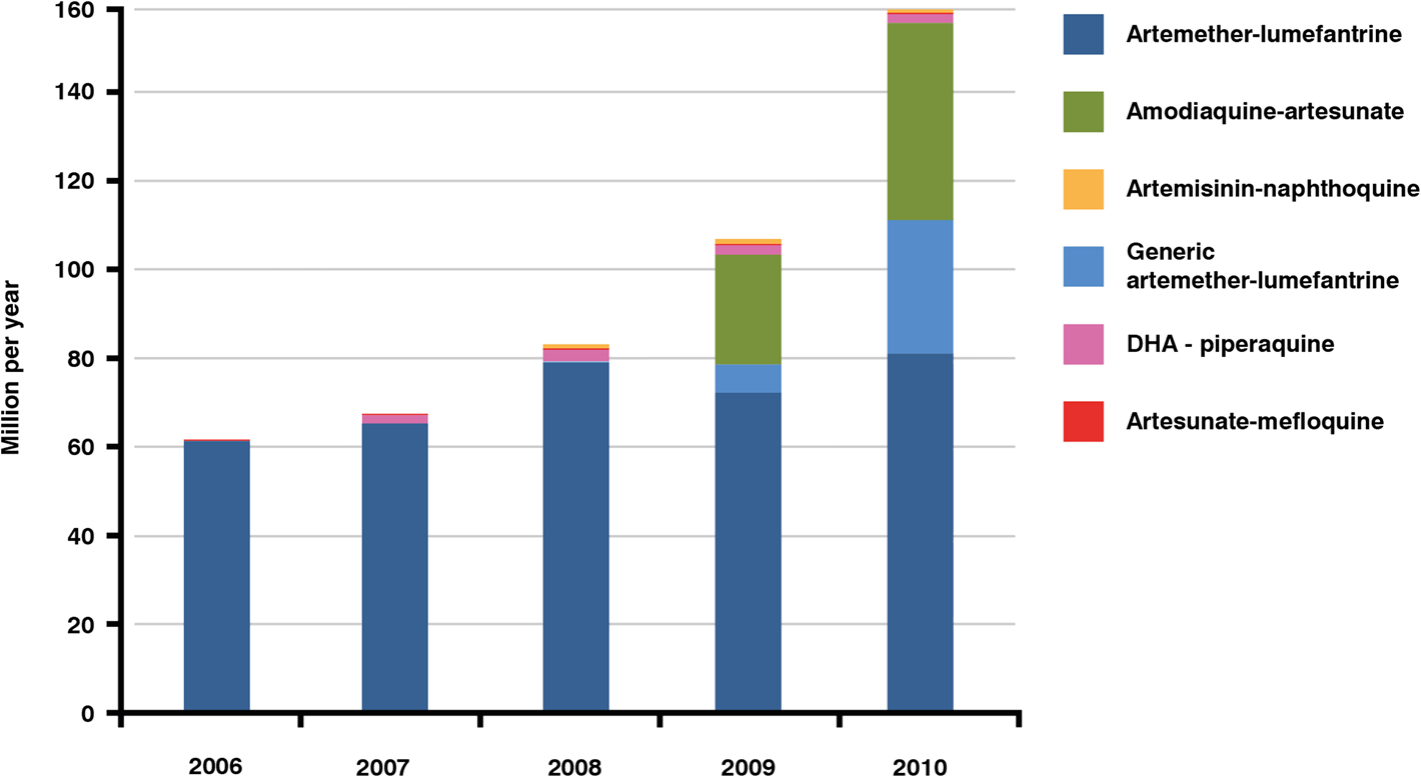
**Sales (USD) of fixed dose artemisinin combination therapy 2006 2010.** These data are compiled from estimates supplied by the WHO-prequalified manufacturers and from data provided by AMFm and include only WHO-prequalified or Global Fund-approved generic versions of the medicines. Sales of DHA/piperaquine have been around two million per year, and naphthoquine artemisinin around one million per year. Mefloquine-artesunate numbers are significantly lower. These numbers compare well with the lower range of the estimates predicted by the Clinton Foundation [42]. The total number of malaria patients was estimated by WHO to have fallen to 225 million in 2009 [7].

Today, first-line medicines against malaria are fixed-dose artemisinin combination therapy (ACT). These medicines are assumed to be active against the blood stages of all of the major forms of Plasmodium which infect humans: *falciparum*, *vivax*, *malariae*, *ovale* and *knowlesi*. Fixed-dose combination therapy has the advantage over co-blistered presentations in that it eliminates the potential for monotherapy [4], which is to be avoided since it risks the emergence and selection of resistant parasites [5]. Six of these have been reviewed by regulatory authorities throughout the world (see Table 1, Figures 1 and 2). Artemether-lumefantrine (Coartem^®^ and Coartem^®^ Dispersible from Novartis), artesunate-amodiaquine (Coarsucam™/Artesunate Amodiaquine-Winthrop^®^ from Sanofi), pyronaridine-artesunate (Pyramax^®^ from Shin Poong Pharmaceuticals) and mefloquine-artesunate from Cipla/DNDi– have been prequalified by WHO [6]. Their launch has had a dramatic impact on the number of courses of treatment available to malaria patients. There has been rapid growth from 62.3 million treatments in 2006 to 159.7 million in 2010 (see Figure 2) although, still, not all medicines reach the patients who need them [7]. Considerable progress has been made on price, with the costs of an adult course of treatment falling to $1.00–$1.40. For infants the price can be as low as $0.30 [8], although in 2011 prices rose due to perceived artemisinin shortages [9]. 

**Table 1:** Fixed - dose artemisinin combination therapy approved or in development (as of November 2011)

**Active ingredients** (**adult dose**)	**Partnership**	**Phase**/**status**	**Product name**(**s**)	**Comments**	**Chemical structure in Figure**1	**References**
Artemether (20 mg), lumefantrine (120 mg) x 4 b.i.d	Novartis, MMV	Launched 2008	Coartem^®^; Coartem^®^-Dispersible	Coartem was launched in 2001. Coartem Dispersible is a specific paediatric formulation with a sweet-tasting flavour. It is given twice per day for three days. Coartem Dispersible decreases gametocytes by 6–8- fold compared to sulphadoxine/pyrimethamine and chloroquine. Approved by Swissmedic in Dec 2008, it received WHO prequalification in Feb 2009. Four dose strengths are registered in 83 countries. In Apr 2009, Novartis received approval from the US-FDA for Coartem, including a ‘Priority Review Voucher’ (PRV).	a), f)	[10-17]
Artesunate (100 mg), amodiaquine (270 mg) x 2 q.d.	Sanofi, DNDi (MMV)	Prequalified 2008	Coarsucam^®^; ASAQ-Winthrop^®^	Marketed as Coarsucam in the private market and ASAQ-Winthrop in the public markets. Given once per day for three days. Originally registered in Morocco, it is now approved in 31 countries (including 25 in Africa) and prequalified in 2008 by WHO, but so far has not been submitted to a Stringent Regulatory Authority. Stability data now available for three years in Zone IVb (30°C and 75% relative humidity), compared to two years for other ACT.	b), g)	[18-23]
Dihydroartemisinin (40 mg), piperaquine (320 mg) x 3 q.d.	sigma-tau, MMV, Chongqing Holley Holding	Approved	Eurartesim^®^; Artekin^®^; Duo-Cotecxin^®^	Available as Duo-Cotexcin since 2005 (approved by Sino-FDA), uses the active metabolite of artemisinin, dihydroartemisinin. Given once per day for three days. Two pivotal clinical trials led to approval by the EMA in 2011. Prequalification expected 2012. Shows superiority to artemether-lumefantrine in post-treatment protection until day 63.	c), d)	[24-29]
Pyronaridine (180 mg), artesunate (60 mg)	Shin Poong, MMV	Pre-registration	Pyramax^®^	Approved by the Korean K-FDA, and by the EMA, article 58 in Feb 2012. Prequalified by WHO. Given once per day for three days. Four pivotal clinical studies were performed 2007–2009, with the first regulatory studies on an ACT in both *Plasmodium falciparum and Plasmodium vivax* malaria. Shows superiority to artemether-lumefantrine in post-treatment protection until day 42.	g), b)	[30-33]
Mefloquine, (200 mg), artesunate (100 mg), x2 qd	Farmanguinhos, DNDi, (Cipla Ltd, MMV), Mepha Ltd	Launched (Brazil) 2008; Launched (Portugal) 2003	ASMQ; Artequin	A fixed-dose combination from Farmanguinhos (Brazil)/DNDi was registered in Brazil in 2008. Given once per day over three days. Use outside Brazil should be accelerated by production by Cipla (India), and Indian registration in 2012. Prequalified by WHO in September 2012. Fixed-dose combination called artequin granules (50 mg artesunate/125 mg mefloquine), originally registered in Portugal (2003) and now sold in Africa in commercial markets.	e), b)	[34-38]
Artemisinin (125 mg), naphthoquine (50 mg) x 8 tablets	Chinese Academy of Military Medical Sciences, Kunming Pharma Corp	Launched (China)	ARCO^®^	Combination of poorly bio-available artemisinin with naphthoquine. Given either as a single dose or split twice within the same day. Clinical studies are not GCP compliant, and report on a limited number of patients. Very little preclinical data available for review. Registered and promoted in more than 10 countries.	a), h)	[39-41]

The two major artemisinin combination therapies are:

(a) Artemether-lumefantrine (Coartem^®^ and the dispersible paediatric formulation Coartem^®^ Dispersible from Novartis). Over 500 million treatments of these medicines have been delivered since the initial launch in 2002, of which over 100 million are now the dispersible form specifically designed for children. Several generic versions of this medicine have now been produced, including those prequalified by WHO.

(b) Amodiaquine-artesunate (Coarsucam™ and Artesunate Amodiaquine Winthrop^®^, from Sanofi). This was initially approved in Morocco, where it is manufactured and was prequalified by WHO in 2008.

There are four other types of ACT prescribed for the treatment of uncomplicated malaria, which are currently at different stages of registration and approval.

(a) Dihydroartemisinin (DHA)-piperaquine (Eurartesim^®^ from Sigma-tau) was approved by the European Medicines Agency (EMA) in October 2011 [43] and is included in the Malaria Treatment Guidelines this year [44]. New data showing two-year stability are now available. Prequalification by WHO and submission in key disease-endemic countries is expected in 2012. Holley-Cotec produce another version of DHA-piperaquine (Duo-Cotecxin^®^) [45], available in many countries, which is expected to be submitted soon for prequalification.

(b) Pyronaridine-artesunate (Pyramax^®^ from Shin Poong Pharmaceuticals) was approved by the Korean Food & Drugs Administration (KFDA) in August 2011 [46], and was approved by the EMA in February 2012, under Article 58 [47], where opinion is given on whether a medicine is suitable for use in countries where the disease is endemic. This avoids the obligation to market the medicine in Europe, and is a decision made in conjunction with WHO, which has now prequalified the product.

(c) Artesunate-mefloquine (ASMQ) is a fixed-dose combination produced by Cephalon/Mepha as a paediatric formulation for commercial markets in Africa, and by the Drugs for Neglected Diseases initiative (DND*i*) in partnership with Farmanguinhos for use in Brazil. From 2012, the fixed-dose combination will be manufactured and registered by Cipla in India, which will accelerate uptake [48]. WHO prequalification was obtained in September 2012. Currently the market price of mefloquine (over $1,000/kg) makes this combination the most expensive ACT, however work is ongoing to lower the cost of manufacturing. A cheaper synthesis of mefloquine has been developed by Development Chemicals in collaboration with DND*i* and MMV. This allows a price similar to other fixed-dose ACT.

(d) Artemisinin-naphthoquine (ARCO^®^, Kunming, China) is available in Africa as a one-day treatment. There are relatively few data available on the efficacy and safety of naphthoquine [49], and the product has not yet been submitted for approval either to a Stringent Regulatory Authority or to WHO. The adult dose of artemisinin is high (15–20 mg/kg) [50], reflecting the poor bioavailability of the parent molecule.

Since over 85% of malaria patients are under five years old, development of child-friendly paediatric formulations remains the priority. Coartem^®^ Dispersible (dispersible artemether-lumefantrine) is the first example of a taste-masked dispersible ACT, and was developed in a collaboration between MMV and Novartis [51]. Two other paediatric formulations are in development: a granule formulation of pyronaridine-artesunate is to be submitted in early 2013 [52], and a dispersible formulation of DHA-piperaquine is planned for submission later in the same year.

Having more than one ACT available is an advantage. Each medicine will have a different impact, depending on the endemicity of the disease, the likelihood of re-infection, diet, co-medications, and the balance between the different forms of malaria: *falciparum*, *vivax*, *malariae*, *ovale* or *knowlesi*. These factors are summarized in Table 2. Resistance to partner drugs has already occurred in some countries, as has been demonstrated for amodiaquine [53], and this remains a threat for other therapy partners in many countries. Clinical signs of decreasing effectiveness of partners have been described, and loss of susceptibility to artemisinin has been reported in the Thai-Cambodia border regions [54,55] as well as more recently in the Thai-Myanmar border region [56]. Having multiple first-line therapies in a country may help reduce the spread of resistance [57,58]. 

**Table 2:** Relative product positioning for the fixed-dose artemisinin combination therapy, highlighting the differences between the medicines

	**Artemether lumefantrine**	**Artesunate amodiaquine**	**Dihydroartemisinin piperaquine**	**Pyronaridine artesunate**	**Artesunate mefloquine**	**Artemisinin naphthoquine**
**Partner**	Novartis, MMV	Sanofi, DNDi, (MMV)	sigma-tau, Pfizer, MMV	Shin Poong, MMV	Farmanguinhos/DND*i*, Cipla; Mepha	AMMS, Kunming Pharma Corp
**Trade name**	Coartem^®^; Coartem-^®^ Dispersible	Coarsucam^®^; ASAQ-Winthrop^®^	Eurartesim^®^	Pyramax^®^	ASMQ	ARCO^®^
**Approval Date**	1Q'01/1Q'09 Swiss-Medic	2Q'07 Morrocco Prequalified 4Q’08	4Q'11 EMA	1Q'12 EMA and Prequalification	2Q'08 Brazil Prequalification anticipated	2005 China
**Stringent Approval**	Yes: CH, US-FDA, WHO Prequalified	No: Morocco, WHO Prequalified	EMA Submitted; approved by the CHMP in Jun 2011, and EU in Oct 2011	EMA Submitted. Approved by the Korean FDA in Aug 2011; approved by EMA in Feb 2012	Farmanguinhos/DND*i* No: Brazil, WHO Prequalification submitted; Mepha product approved in West Africa and Portugal	No: Sino-FDA for the moment
**Formulation**	Tablet (adult), dispersible flavoured tablets (child)	Dispersible tablets for all ages	Tablet (adult, child), dispersible formulation for children (submission in 2014)	Tablet (adult), sachet with granules (child), (submission in 2014)	Tablet (adult, child)	Tablet (adult, child)
**Key Strengths**	350 million treatments to date. Paediatric formulation is dispersible and flavoured	First line therapy in francophone Africa. Three-year shelf life. Once per day, three days. Paediatric dose.	Once per day, three-day treatment course. Long terminal half-life of piperaquine. Strong post-treatment protection of 42 days	Once per day, three-day treatment. Granulated paediatric formulation with taste-masking. Best preclinical and clinical data against *Plasmodium vivax*.	Once a day, three-day treatment. Strong post-treatment prophylaxis. Active against chloroquine resistant *P. vivax*.	Single dose treatment, may be split over two days.
**Key Weakness**	Twice per day	Amodiaquine resistance in some countries. GI adverse events	Previous concerns on stability, but data now support two years under Zone IVb conditions	Tablets can only be given to patients >20 kg. Limited repeat dose data. Currently only recommended for a single treatment	Psychiatric and GI adverse events. Mefloquine-resistant strains exist. Concern over use where mefloquine prophylaxis is recommended. Currently relatively expensive ($2.50)	No GCP clinical studies or safety data. Insufficient information on ARCO for children. No stringent regulatory approval
**Market size & strength 2010**	First line treatment in 35 countries. 82 million treatments (37 million Coartem-^®^ Dispersible) per year. Estimated 21 million are generic artemether/lumefantrine.	45 million (fixed-dose combinations). First line treatment in 17 countries (West Africa)	Two million treatments per year. Registered in 30% of African countries. First line therapy in Cambodia, on treatment guidelines in six countries	Not launched yet. Should be medicine of choice in Asia/Pacific dual-infection areas.	Still relatively small (200,000 treatments). An Indian approval would significantly change this.	Registered in more than 10 countries. Treats one million patients per year.
**Stability**	24 months	36 months	24 months	24+ months	36 months (Brazil)	Unknown
**Public sector pricing** (**USD**)	$0.74 weighted average (from $0.37-$1.41 depending on weight)	$0.60 weighted average (from $0.30-$1.50 depending on weight)	Estimated $0.90-$1.10 weighted average (from $0.50-$1.50 depending on weight). Final price may be higher due to DHA prices	$1.10 weighted average (from $0.34-$2.12, lowest to highest weight band). Final price may be higher due to artesunate prices	Currently at $2.50 per adult. New mefloquine synthesis should make the price similar to other ACT	Not sold in the public sector.

Pyronaridine-artesunate is currently the only ACT with regulatory approval for activity against *P. vivax*, although it is widely assumed from field experience that other fixed-dose combinations will be equally active against *P. malariae* and the blood stages of *P. vivax* and *P. ovale*[31,32].

**Figure 3:**
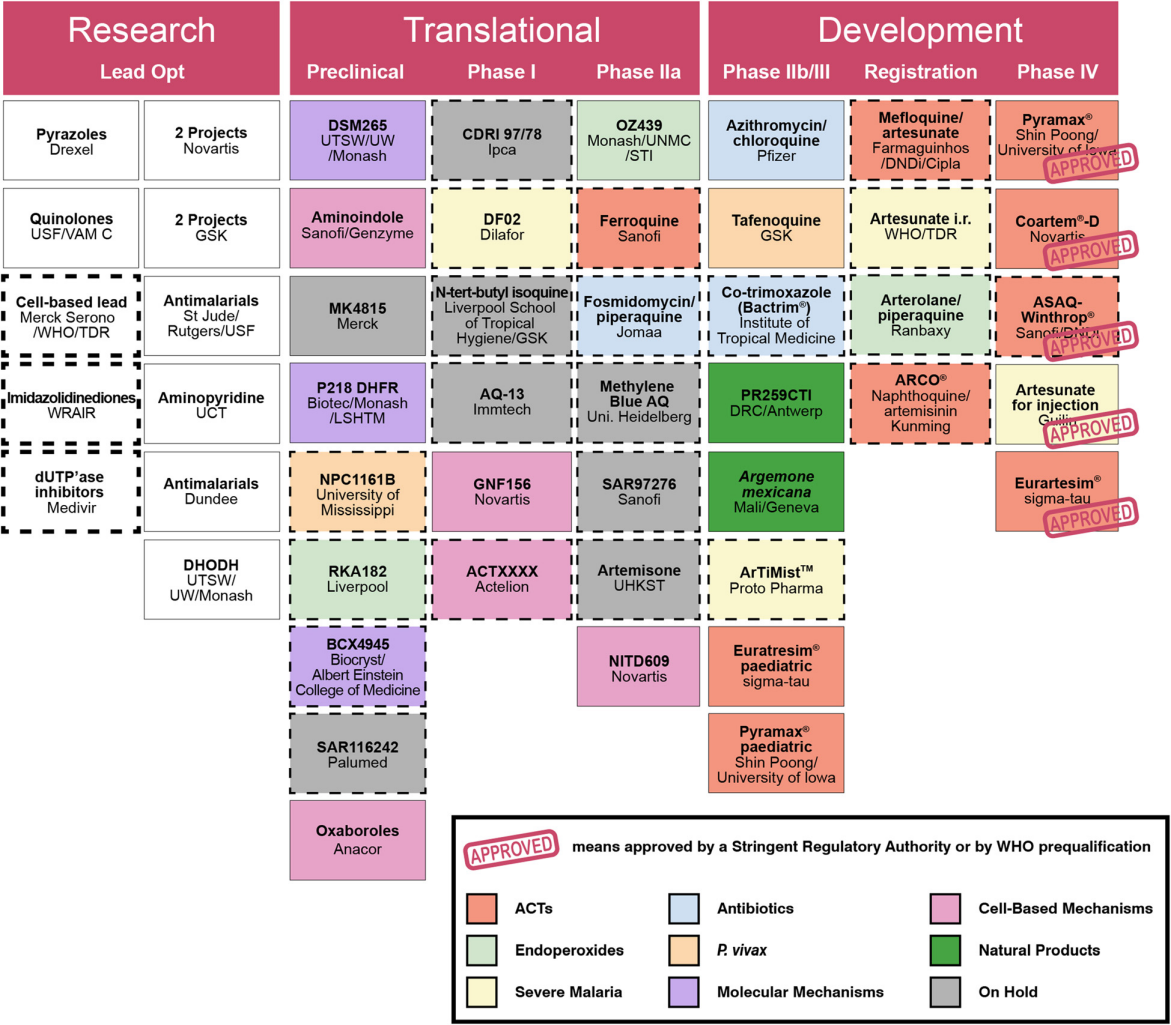
**The global portfolio of anti malarial medicines under development organized by development stage (as of March 2012).** This includes all projects in formal regulatory preclinical safety and pharmacokinetic studies. Projects carried out in collaboration with MMV are shown in open boxes, whereas those with no active MMV involvement are shown with a dashed border. Data are from MMV internal reports [59], and Thomson Pharma. Compounds have been defined as ‘on hold’ when no significant progress along the development process has been made publicly available in the last 12 months. Natural products are defined as Herbal Medicinal Products undergoing testing in malaria patients in GCP quality studies, using standardized extracts. Updates of this figure are available on a quarterly basis [60]).

**Figure 4:**
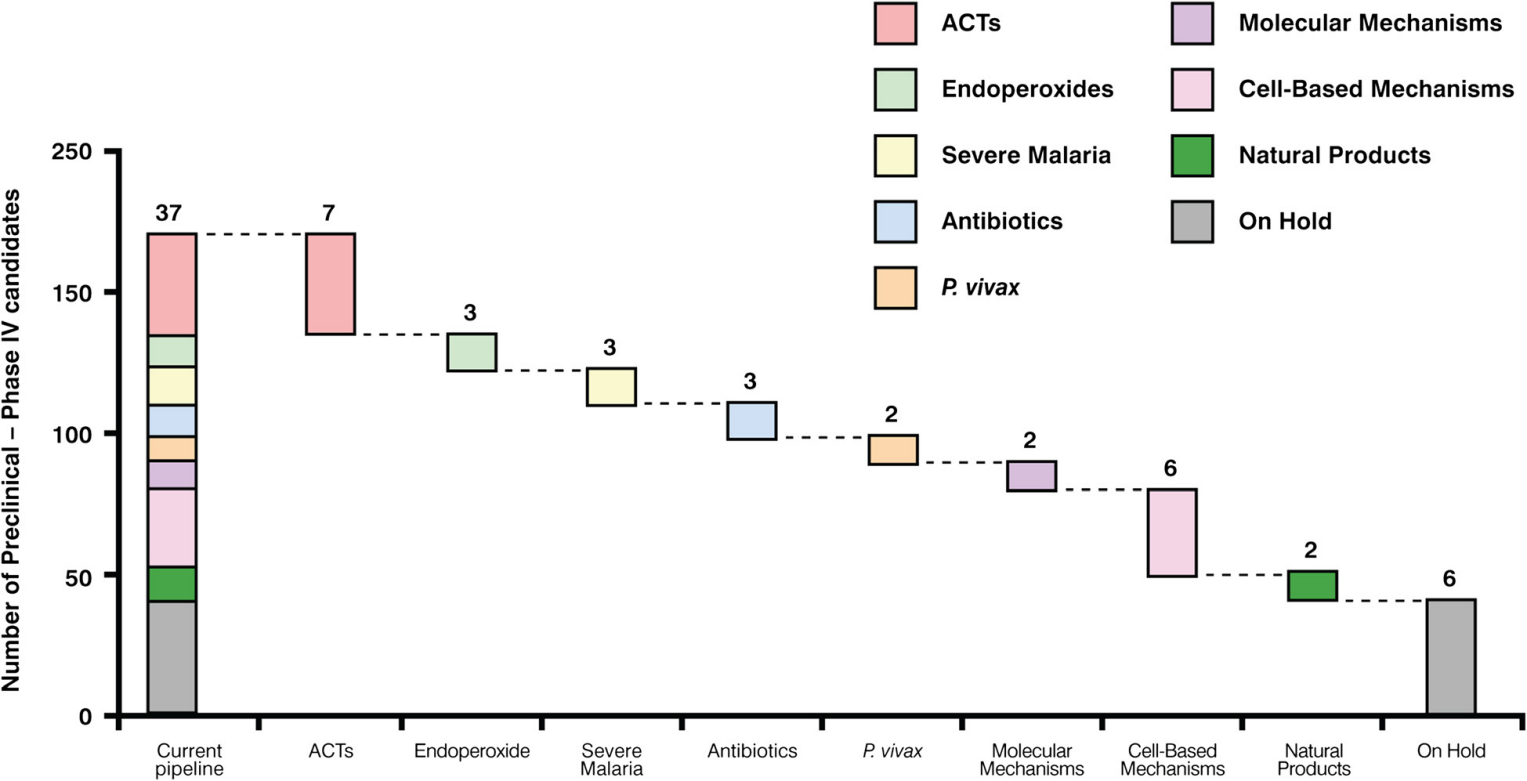
**The global portfolio of anti malarial medicines organized by therapeutic type.** Compounds have been defined as ‘on hold’ when no significant progress along the development process has been made publicly available in the last 12 months. This analysis is important to show the emergence of new classes of medicines. Updates of this figure are available on a quarterly basis [60]).

What challenges remain for the next generation of medicines? There is a continual threat of the emergence of resistance, both to artemisinin and the partner medicine. This will require new classes of medicines. In addition to this there are four areas of focus for drug discovery. Firstly, in the context of malaria eradication, there is a need for medicines that can be administered as a single dose, which will allow direct monitoring of administration and improve compliance. These should have activity against all existing resistant strains of parasite. Secondly, new medicines are needed that kill gametocytes, and thus prevent transmission. Thirdly, there is a need for medicines which prevent relapses of *P. vivax*. Finally there is a need for molecules with longer half-lives to give chemoprophylaxis or long-term protection against re-infection [58]. Figure 3 highlights the global portfolio of anti-malarial medicines in development organized by development stage (as of March 2012). Figure 4 shows the global portfolio of anti-malarial medicines organized by therapeutic type, as discussed below. 

### New treatments for severe malaria

In 2010, a study in African children with severe malaria [61] showed a significant reduction of mortality using intravenous or intramuscular injections of artesunate in place of quinine, confirming earlier results in adult patients from Asia [62,63]. WHO now recommends artesunate as a first-line therapy in severe malaria [44]. Artesun^®^(a for injection), manufactured by Guilin Pharmaceuticals (see Table 3), was the first to be prequalified by WHO in 2010 [6]. The cost of approximately $1.00 per treatment is justified by the better outcome compared with quinine [64]. As with ACT, it is important that there are multiple and reliable production sources, otherwise countries are reluctant to change treatments. IPCA manufactures artesunate for injection for India (see Table 4), and is planning to submit for prequalification. 

**Table 3:** Products under development for severe malaria, or as artemisinin monotherapy

**Active ingredients**	**Partners**	**Phase**/**status**		**Comments**	**References**
Intra-rectal artesunate	UNICEF-UNDP-World Bank-WHO Special Programme for research and training in Tropical Diseases (WHO-TDR)	Pre-registration		Intra-rectal artesunate improves outcomes in sub-group in retrospective analyses: patients >6 hours from hospital, and <6 years old. No manufacturing or distribution partner has been defined.	[65-67]
Sevuparin sodium (DF-02)	Dilafor AB	Phase IIa		Heparin analogue with a low anticoagulant activity. Phase I data were reported in Oct 2009 with doses as high as 420 mg. A Phase II study started in 2011	[68]
Artesunate for injection	Guilin Pharmaceutical	WHO prequalified 2010		Artesunate for injection has been produced since 1987. In 2010, Guilin gained WHO prequalification (with support from MMV). The clinical data in both the Aquamat and Seaquamat studies used Guilin material. Current price is $1.40 per 60 mg vial, over 2 500 000 vials sold in 2011	[69]
ARH1 (150 mg/ml artemether)	Lincoln Pharmaceuticals	Launched (India) 2011		ARH1, an injectable formulation of arteether (ethyl ether derivative of artemisinin). It is oil soluble, has a long elimination half-life (greater than 20 hours), and is more stable than other artemisinin compounds: launched in India in Jun 2011.	[70]
SAR97276 (albitiazolium bromide)	Sanofi/CNRS	Phase II		Bisthiazolium choline uptake inhibitor, not orally bio-available. Being developed as an intramuscular injection for severe malaria. Initial Phase II data showed relatively poor efficacy in uncomplicated malaria, and may require a higher dose. Medicine could have the advantage of being shipped in pre-filled syringes, making delivery simpler.	[71] NCT00739206
Artemether	Eastland Medical, Star Medical, Protopharma Ltd	Phase III	ArTiMist™ (sublingual mouth spray)	An artemether sublingual mouth spray. A Phase II study was conducted to compare the efficacy of ArTiMist™ and intravenous quinine in children infected with severe malaria, or uncomplicated malaria and gastro-intestinal complications (NCT01047436). The difficulty is to make sure this product does not break WHO’s call for the withdrawal of artemisinin monotherapy in uncomplicated malaria.	NCT01047436 NCT01258049
E6446 (TLR9 antagonist)	Eisai Co Ltd	Preclinical		Toll-like receptor antagonists. TLR 9 antagonism has been suggested to suppress cerebral vascular lesions and leakage of vascular contents. The difficulties in interpreting the murine data mean additional proof of mechanism in humans will be needed before starting a severe malaria trial.	http://www.eisai.com

**Table 4:** New products under development

**Active ingredients**	**Partnership**	**Product name**(**s**)	**Phase**	**Comments**	**References**
Azithromycin 250 mg, chloroquine 155 mg	Pfizer, MMV, London School of Hygiene and Tropical Medicine	AZCQ	Phase III	Azithromycin is a macrolide requiring 2000 mg/day to be effective as monotherapy but shows clinical synergy with chloroquine even where chloroquine resistance is as high as 50%. Entered Phase III for intermittent preventive treatment of *Plasmodium falciparum* malaria in pregnancy (IPTp) in Oct 2010. The primary outcome is a reduction in the number of subjects with suboptimal pregnancy outcome, and results should be available in 2014.	[72-76]
Trimethoprim/sulphamethoxazole	Studies by Institute of Tropical Medicine, Antwerp in Zambia and UCSF in Uganda	Co-trimoxazole	Phase III for malaria	An antibacterial with activity against malaria. Early data in children showed similar efficacy to amodiaquine artesunate. A Phase III study comparing against SP and DHA-piperaquine is expected to be completed in Jul 2014. A Phase III study with 1,714 subjects in Zambia is testing its effectiveness as prophylaxis to prevent malaria in pregnancy, expected to be completed in Nov 2011	[77-80] NCT01053325 NCT00948896
Rbx11160/OZ277 150 mg, piperaquine phosphate 750 mg	MMV, Ranbaxy	Arterolane maleate + piperaquine phosphate	Phase III	First generation synthetic peroxide (trioxalane), given as one tablet per day for three days. Arterolane as a monotheraphy drug showed lower efficacy than artesunate with seven days of dosing. A Phase III trial in India, Bangladesh and Thailand is now underway. The medicine was approved in India in early 2012.	[81-86] NCT00362050
OZ439	MMV, (University of Nebraska, Monash University and Swiss TPHI)		Phase IIa (monotherapy)	Next-generation synthetic peroxide. Phase I showed OZ439 was safe at doses up to 1,600 mg as a single dose, and gives plasma concentrations with anti-parasite activity up to 72 hours. A Phase IIa study in *P. falciparum* and *P. vivax* patients was completed in mid-2012.	[84] NCT01383096 NCT00928083
SSR97193, artesunate	Sanofi	Ferroquine	Phase IIa	4-aminoquinoline active *in vitro* against multidrug resistant *P. falciparum*. Phase II studies showed that it is active in combination with artesunate in African adults and children. Project was put on hold, presumably because of insufficient product differentiation.	[87-91]
Fosmidomycin, clindamycin	Jomaa Pharma Ltd	Fosclin	Phase IIa	Fosmidomycin is a DOXP inhibitor, used in combination with the antibiotic clindamycin. A study in children showed good tolerability and 94% efficacy. A further Phase II was completed in Sept 2011.	[92-96]
Methylene blue, chloroquine	Ruprecht-Karls-Universität, Heidelberg, DSM		Phase II	Methylene blue was originally suggested by Paul Ehrlich. The combination MB-CQ is safe in adults and children with or without G6PD deficiency. Local acceptability studies imply discolouration of urine is not seen as a major issue.	[97-99]
NITD609	Novartis, MMV		Phase IIa (monotherapy)	Spiroindolone suppresses protein synthesis in the parasite, working through PfATP4. NITD609 has the pharmacokinetic properties compatible with once-daily oral dosing for the potential treatment of falciparum and vivax malaria. A Phase IIa trial started in December 2011.	[100,101]
Actelion antimalarial	Actelion		Phase I	A compound of undisclosed structure with similar parasite reduction rates as chloroquine. Completed Phase I in summer 2012	
GNF156	Genome Foundation of Novartis, MMV		Preclinical	An imidazolopiperazine active against blood, liver and gametocytes, with stages of *Plasmodium*, and active in murine models. Preclinical safety studies started in the first quarter of 2011, and Phase I early in 2012.	
Oxaboroles	Anacor, MMV		Preclinical	A new class of oxaboroles. Highly potent against *P. falciparum* parasites, and curative in animal models. Has a good safety profile and excellent drug-like properties, and low cost-of-goods.	
DSM265	University of Texas Southwestern, MMV, University of Washington, Monash University		Preclinical	A dihydroorotate dehydrogenase (DHODH) inhibitor. In June 2010, a compound was chosen from the inhibitor series for full preclinical development. First in human studies are planned for early 2013.	[102-105]
MK4815	Merck, MMV		Preclinical	A cell-based inhibitor that has demonstrated potency against *P. falciparum* malaria when tested *in vivo*. Its mechanism of action appears to involve the mitochondrial electron transport chain of the parasite, and when administered, concentrates in infected erythrocytes. Due to safety issues, the molecule is still in preclinical evaluation.	
P218	BIOTEC, Monash University, LSHTM, MMV		Preclinical	A dihydrofolate reductase inhibitor binds with high affinity to the wild-type and resistant DHFR enzymes and has an excellent ADME-PK profile. Currently in preclinical development, although there are concerns about the impact of pre-existing DHFR mutant stains of parasite.	
Genz668764	Genzyme Corp (Sanofi), MMV		Preclinical	An aminoindole, with activity against both P. falciparum and P. knowlesi. This family has the advantage that it has been impossible so far to produce resistant mutant strains of parasite. Preclinical evaluation started in early 2011.	[106]
RKA 182	Liverpool School of Tropical Medicine, Liverpool University		Preclinical	Lead compound from a series of tetraoxanes with rapid anti-parasitic activity. Has shown anti-malarial activity against a range of multi-drug resistant isolates. Its half-life is intermediate between OZ277 and OZ439. Next-generation compounds are currently being tested.	[107,108]
BCX4945	Albert Einstein College of Medicine, Biocryst Pharmaceuticals,		On Hold	Parasites cultured in human erythrocytes can be killed rapidly by this purine nucleoside phosphorylase (PNP) (immucillin-H). Purine salvage pathways in the mouse suggest a mechanism for lack of *in vivo* activity, and that these compounds will have to be tested directly in human challenge models.	[109,110]
CDRI 99/411	Ipca, CDRI		On Hold	A trioxane anti-malarial candidate, shown initially to be safe in pre-clinical studies. An IND was approved in March 2010 in India, but no further progress has been reported.	[111]
AQ-13	Immtech		On hold	AQ-13 is another 4-aminoquinoline, active against chloroquine-resistant *P. falciparum* infection. A Phase I study with 126 individuals was successfully completed in 2005, but no further development was reported. The compound has no significant advantages over other 4-aminoquinolines.	[112,113] NCT00323375
N-tertiary-butyl isoquine	Liverpool School of Tropical Medicine, GSK, MMV		On hold	N-tert-butyl isoquine was developed as a safer alternative to amodiaquine. In May 2008, a single-blinded, placebo-controlled, randomized Phase I trial was initiated in healthy volunteers. The trial was stopped later in 2008 as a result of adverse events, at the highest dose. Experiments are ongoing to recalculate the estimated human effective dose.	[114-117]
SAR116242/PA1103	Palumed, MMV		On hold	A fusion compound containing a trioxane ring and a 4-aminoquinoline group. It entered preclinical development in 2007, but has not progressed into human studies.	[118]
Artemisone	Hong Kong University of Science and Technology, (MMV, Bayer)	Artemefone (semisynthetic artemisinin derivative)	On hold	In preclinical studies, artemisone, a new artemisinin derivative proposed to have enhanced safety over artesunate. Currently being discussed as an option for treating artemisinin-resistant malaria, although clinical trials have not started, and a commercial partner is still being sought.	[119-122] NCT00936767

Other therapies for severe malaria are in the pipeline. Most are adjunctive therapy, given on top of a schizonticide to reduce the sequelae of severe malaria, such as neurological damage. The track record of most potential adjunct therapy has not been compelling, despite promising results in rodent studies. Adjunct approaches have generally shown little benefit. Recent reports on molecules such as N-acetyl cysteine [123] and pentoxifylline [124] failed to show any significant benefit. One interesting example of adjunct therapy is DF-02 (sevuparin sodium), a heparin derivative with no anti-coagulation activity that blocks rosetting (a key event in severe malaria [68]), which has just completed Phase I trials. The only other molecule specifically being developed for this indication is a TLR-9 antagonist (E6446) from Eisai [125,126]. One success has been sublingual sucrose, which overcomes hypoglycaemia and, in a pilot study, resulted in a significant reduction in mortality [79].

The only new schizonticide specifically being proposed for severe malaria is SAR97276 (albitiazolium bromide), a choline antagonist [71,127]. It is being positioned in severe malaria because of its poor oral bioavailability. Phase II trials have shown higher doses are likely to be needed, at least for uncomplicated malaria [128]. However, given the size of the clinical trials required to show at least non-inferiority regarding mortality compared to artesunate, no new product is likely to be developed for severe malaria unless artesunate becomes compromised by resistance in many countries.

The remaining medicines for severe malaria are all formulations of artemisinin derivatives. An intra-muscular artemether is included in WHO’s treatment guidelines and is produced by Kunming and Sanofi, amongst others (Table 3). No prequalified drug is available yet though, and there is a question mark over neurotoxicity as seen in preclinical trial animal species, but never confirmed in humans [129]. A sublingual spray of artemether from Eastland Medical Systems Ltd/Proto Pharma is being developed, but there is the risk that this product is most likely to be used as monotherapy treatment of uncomplicated malaria, in contradiction to WHO guidance. Finally, the UNICEF-UNDP-World Bank-WHO Special Programme for research and training in Tropical Diseases (WHO-TDR) is developing artesunate suppositories as pre-referral treatment for patients with severe malaria, enabling them to receive at least some treatment prior to arrival at hospital. Clinical studies involving 12,068 patients showed that this increased the probability of survival of patients more than six hours away from hospital and for the youngest patients (100 mg dose) [66]. This product still needs to be approved by a Stringent Regulatory Authority and/or WHO prequalification, and will require a partner for manufacture and distribution. Adequate distribution of these suppositories to health centres more than six hours away from hospital will pose a significant challenge.

### New products in pregnancy

In areas of high transmission, such as sub-Saharan Africa, malaria in pregnancy is a key cause of maternal, perinatal and neonatal morbidity. Case management of malaria seems possible with ACT in the second and third trimesters. Use of artesunate in the first trimester is contraindicated because of the side effects seen in preclinical safety models [130], although these effects have not yet been seen in the registries of patients who were accidentally treated with ACT during pregnancy [10]. The correct dosing of ACT in pregnancy needs to be better defined, as studies show that current doses may be inadequate [131-133]. As well as treatment, medicines may be used for intermittent preventive treatment in pregnancy (IPTp) to reduce infections and improve pregnancy outcomes. Options for therapy include azithromycin-chloroquine, mefloquine and also DHA-piperaquine (Table 4). Azithromycin has poor activity against malaria, but synergizes with chloroquine, such that the combination kills even chloroquine-resistant strains [75]. Similar clinical synergy has been seen between azithromycin and quinine [74]. This combination is antibacterial, potentially reducing the risk for new-borns and mothers from sexually transmitted bacterial infections [134]. Studies are on-going with another antibiotic combination, co-trimoxazole (sulphamethoxazole-trimethoprim), in HIV co-infected women, by the Institute of Tropical Medicine, Antwerp and University of California, San Francisco, USA (NCT00948896).

The other medicine commonly discussed for IPTp is mefloquine, which shows significant clinical benefit [135]. Two complications are side effects and cost. Mefloquine causes nausea and neuropsychiatric disorders amongst Caucasian volunteer cohorts [136], but studies show that side effects are balanced by the positive influence of the drug [37]. On-going efforts sponsored by MMV have optimized mefloquine synthesis, with the potential to lower the cost-per-treatment more than two-fold to less than $400/kg. The use of ACT in IPTp is complicated by concerns over the use of artemisinin in pregnancy, and also the concern over using the same medicines for prophylaxis and treatment.

### Next generation endoperoxides

The world demand for artemisinin products is large. Treating all malaria cases could require in excess of 200 tonnes of artemisinin per year. The price of artemisinin from plants has fluctuated widely (between $300/kg and $1,500/kg), and material can take up to two years from order to delivery. This creates a problem for low-cost therapy. Three solutions have been proposed: first, the use of higher-yielding seeds [137]; second, using yeast for scalable production in bioreactors [138], where Sanofi plans first commercial tonne-scale production in 2012; and third, to make synthetic endoperoxides.

MMV established a project with the University of Nebraska, the Swiss Tropical and Public Health Institute, and Monash University to develop synthetic endoperoxides in 2000. The first clinical product was OZ277 (now called Rbx11160 or arterolane). This showed activity in Phase IIa trials [139] in uncomplicated falciparum malaria. However, the clinical activity was not as good as artemisinin, showing adequate clinical and parasitic response at day 28 (ACPR28) of 60–70% after seven days’ treatment, compared with a 95% response with artesunate. The plasma exposure was non-linear above 100 mg, and OZ277/Rbx11160 was unstable in infected blood (presumably due to free ferrous iron) [84]. Despite this, Ranbaxy have completed a Phase III study of OZ277/Rbx11160 (150 mg) and piperaquine (750 mg), and the product has been approved in India. This is the first medicine to be developed in India. The lower efficacy of OZ277/Rbx11160 compared to artesunate [84] may increase the pressure for resistance against piperaquine, which is already being reported in Cambodia [140]. MMV’s next-generation clinical candidate, OZ439, has superior pharmacokinetics: exposure is dose-proportional, similar in patients and volunteers, and plasma concentrations remain above the mean parasiticidal concentration for more than 72 hours after a single dose, suggesting it could be part of a single-dose cure [84]. Phase IIa studies in uncomplicated *P. vivax* and *P. falciparum* malaria have now been completed. The next stage is to investigate drug interactions in volunteers with potential partner drugs.

Three other synthetic endoperoxides have been in preclinical development (Table 4). RKA 182 is a tetraoxane, from the Liverpool School of Tropical Medicine and the University of Liverpool, with a simpler, more symmetrical molecule that may have a lower cost-per-treatment. Pre-clinical testing showed it to be superior to OZ277 [107], and further work is ongoing to identify a molecule with pharmacokinetics similar to OZ349. CDRI 99/411 [141] from the Central Drug Research Institute in India was taken into Phase I by IPCA but the project is currently on hold. A trioxalane (a six-membered endoperoxide ring), fused with a 4-aminoquinoline, was produced by Palumed (PA1103/SAR116242) [118], but was abandoned in preclinical development. Finally, the semi-synthetic endoperoxide, artemisone, showed good activity in Phase II studies in 2009 [121]. Potential advantages of artemisone include lower dose and potential activity in artemisinin-resistant malaria (given the additional thiomorpholino ring). Initially the compound was developed by Bayer, however the company is no longer involved with this molecule and a new partner is needed before clinical studies can recommence.

### Preventing the relapse of *Plasmodium vivax* malaria

The current gold standard for preventing relapse in *P. vivax* or *P. ovale* is primaquine, an 8-aminoquinoline [142]. There are two major issues with its use: 14 days’ treatment is required, reducing compliance to close to zero [143], and there is an elevated risk of haemolysis in patients with glucose-6-phosphate dehydrogenase (G6PD) deficiency [142], present in 10–20% of the population in malaria-endemic areas [144].

The only molecule currently in clinical development for preventing relapses is tafenoquine (WR 238605), another 8-aminoquinoline originally developed by the Walter Reed Army Institute of Research. Tafenoquine has a longer half-life in man, with potential as a single-dose treatment [144-146]. The clinical programme is being partnered by GSK and MMV and has now started recruiting patients for a Phase II dose-finding study. Initial results on both the relative efficacy and safety in G6PD-deficient subjects, compared with chloroquine alone or compared to primaquine, are expected towards the middle of 2013. NPC1161B is a related 8-aminoquinoline from the University of Mississippi [147], active against relapses in primate models. At this stage, it is difficult to find any superiority of NPC1161B over tafenoquine, apart from its effect on insect stages [148], highlighting the need for a preclinical model for G6PD-dependent erythrocyte deformation and haemolysis.

### Other products in clinical development

The remaining projects in development fall into several distinct groups:

(a) **Next-generation aminoquinolines.** There are a number of 4-aminoquinolines or amino-alcohols in development. These are positioned as the next-generation molecules after the current ACTs. As there are already six artemisinin combination products with some form of regulatory dossier, and all have ACPR28 >95%, this makes the hurdles extremely high for such new molecules. A prime advantage would be to have molecules with better safety profiles, but it is almost impossible to predict this pre-clinically. A lower clinical dose could be an advantage (total doses of 1,920 mg lumefantrine, 1,620 mg amodiaquine or 2,880 mg piperaquine are required over three days for adults [44]), especially if this could be given as a single dose. Phase II studies have been completed on ferroquine (from Sanofi) in combination with artesunate, where total doses as low as 300 mg have shown clinical activity (see Table 4). Naphthoquine (from Kunming) is used in a dose of only 400 mg in the combination with artemisinin, and both of these compounds show some promise. Two other aminoquinolines are on hold: AQ-13 is a modified chloroquine, showing similar exposure in Phase I [147], but which is not sufficiently differentiated. N-tertiary-butyl isoquine is a modified amodiaquine from the University of Liverpool and the Liverpool School of Tropical Medicine [115,116], which has not been progressed since the end of Phase I studies.

(b) **Antibiotic combinations.** In addition to the azithromycin-chloroquine and sulphamethoxazole-trimethoprim combination discussed above in the pregnancy section, two other antibiotic-containing combinations are being explored (see Table 4). The 1-deoxy-D-xylulose 5-phosphate (DOXP) inhibitor, fosmidomycin, has been developed with the lincosamide antibiotic, clindamycin, by Jomaa Pharma GmbH (NCT00217451). Fosmidomycin appears to have a rapid parasite-killing activity in adult patients, but not in children, although a full time-course has not been published, and the dose of 3,600 mg/day may ultimately be problematic [149]. Antibiotic combinations could be considered as fall-back therapy in the event that artemisinin resistance becomes a significant clinical issue. Under these circumstances, the standard of care could become seven days quinine plus antibiotic, and therefore the barrier to success would be lower. However, as new classes of molecules enter clinical development from discovery, the relative importance of these antibiotics decreases. The major challenge of effectively managing a portfolio of anti-malarial medicines is to balance the investment in old classes of molecules with that in new molecules with unknown risks and opportunities.

(c) **New compounds against molecular targets.** Two compounds against molecular targets have also moved into development. The enzyme dihydroorotate dehydrogenase (DHODH) is known to be essential for the survival of the parasite. A new inhibitor, DSM1, was identified from high-throughput screening at the University of Texas, and the three-dimensional structure of the enzyme-inhibitor complex has been resolved [103]. An improved version, DSM265 has recently entered preclinical development. A next-generation inhibitor of dihydrofolate reductase, P218, entered preclinical development, and work has been focused on allaying fears about the propensity to select for pre-existing mutations in dihydrofolate reductase (DHFR) from the widespread use of pyrimethamine [150]. BCX4945, an inhibitor of purine nucleoside phosphorylase developed for other indications, has been suggested for use in malaria [110]. However, the compound showed no activity in mouse models.

(d) **New compounds from cellular screens.** Despite these success stories, screening ‘validated’ molecular targets has not been particularly fruitful over the past few years, an experience shared with antibacterial drug discovery [151]. Screening directly against the whole parasite has, therefore, been prioritized. Over the past few years, six million compounds (from over 20 companies and university groups) have been screened against the parasite in the intra-erythrocytic stages. This has resulted in almost 30,000 (0.5%) compounds identified with activity at micromolar levels [152-154]. This rate is higher than predicted, and also is higher than that seen when screening molecular targets. The first compound to be developed from the parasite-focused screening is NITD609, a spiroindolone developed by the Novartis Institute for Tropical Diseases in Singapore as part of a collaboration with the Swiss Tropical and Public Health Institute and the Dutch Biomedical Primate Research Center [101]. This is now in Phase IIa clinical studies. The move from screen to Phase IIa within five years is a tremendous achievement for a new class of molecules. Target identification in parallel to lead optimization suggested the P-type cation-transporter ATPase 4 (PfATP4), which interestingly had been characterized, but not prioritized as a target [155]. The second compound from this collaboration is GNF156, an imidazolopiperazine identified by the Genome Foundation of Novartis in San Diego as part of the same collaborative network. It exhibits *in vitro* potency against blood, liver schizont and gametocyte stages of *Plasmodium*, (but not the hypnozoites), is orally efficacious in mouse malaria models [156], and has recently entered human volunteer studies.

Several other molecules coming from similar screens are entering development. Novel oxaborole-containing compounds synthesized by Anacor have been identified by screening their library against *Plasmodium*. Genzyme identified an aminoindole in a project with the Broad Institute in Boston [106], which is particularly interesting since no resistant parasite strains have yet been identified. Merck identified MK4815, also based on cellular assays, which underwent preclinical evaluation with MMV and has not progressed further due to a narrow safety window. Finally, the Swiss biotechnology company Actelion identified an anti-malarial from a parasite screen of a highly focused set of molecules, and this compound is also in Phase I clinical trials.

(e) **Other compounds.** Methylene blue has long been known to have an activity against malaria [99], presumably by changing the redox potential of the infected cell. Phase IIa studies have been carried out with other compounds in combination with chloroquine. The results are interesting, but the combination therapy is less active than ACT [98]. Recent suggestions of activity against the gametocytes may require a re-evaluation of its clinical utility.

### Natural products

There is a substantial interest in natural products and the possibility for their role as new anti-malarials. This is not a new departure: three of the mainstays of malaria treatment come from natural products: quinine, lapachol (which led to atovaquone) and artemisinin. The Chinese method *dao-xing-ni-shi* – ‘acting in the reversed order’ – was used by Chen Guofu [157] to investigate herbal extracts used by a population as their anti-malarial treatment. However, many of the extracts known to ‘cure’ malaria have never been observed in situations where (i) it is clear that the patients had malaria (defined by WHO as fever and parasites); (ii) how much of the extract they consumed; and (iii) whether they had malaria seven or 14 days later. In addition, given that most African adults have some sort of immune protection against the malaria parasite, a placebo rate of around 70% is possible, confounding the studies.

The identification of new active natural products in malaria has been disappointing [157]. There have been relatively few new compounds identified by purifying individual natural product molecules from extracts and screening them individually against the parasite. This has led to a call for using molecules in combination [159], or for a return to more observational clinical studies [160].

To date, analysis of the literature has found few natural product extracts in development where the human data are clear. One is an extract of *Nauclea pobeguinii*, a plant from the Democratic Republic of Congo, which showed a parasite and clinical response of 90.3% at day 14 in 65 patients in Phase II [161]. Although this is below the WHO threshold, it is a much better result than would be seen with either a quinine or artemisinin extract. The second natural product is an extract of *Argemone mexicana* (Mexican poppy) from Mali, which shows a cure rate of 89% at day 28 in Phase II studies [162]. In both cases, the challenge is to produce standardized extracts for clinical studies. Eventually standardized extracts could become treatments by themselves, similar to the Indian Ayush-64, the Ghanian Phyto-laria, and the standard treatment (*Argemone mexicana*) in Mali [158]. Moreover, if the active ingredient was identified, this could be developed into a standardized form. There is a strong likelihood that the active ingredient will be a metabolite of the original extract, as these extracts are relatively weak in *in vitro* assays. Therefore, one priority is to analyse the human metabolism of these interesting extracts.

### Significant challenges ahead: changing priorities for the eradication agenda

The announcement of a malaria eradication agenda and its subsequent elaboration by various working groups has helped identify priorities for the future [1]. Beyond that, new medicines would ideally fit several TPPs (for more details please see [3]).

(a) **Single-dose cure.** Not only would changing from a three-day course of therapy to a single dose decrease the cost of treatment but also it would enable directly observed administration, making it far less easy for patients to take an incomplete course of treatment. Medicines that are safe enough to be given as a single dose are needed, that stay at the site of action for at least two parasite life cycles. These medicines need to be active against all species of malaria, especially drug-resistant strains. In addition, it must be clear that the compounds do not easily select for resistant phenotypes in strains that have not yet emerged. The difficulty of studying artemisinin resistance is the current lack of stable cell assays and molecular markers, which make it almost impossible to characterize drug action.

(b) **Transmission-blocking.** Preventing the parasite from being transmitted back to the mosquito in a blood meal would break the cycle of transmission. Compounds are being sought that are active against the gametocyte stage, particularly stage five. In the next few years, it is hoped that high-throughput screening will be available to screen larger collections of molecules specifically for this task. Understanding the activity of compounds against *P. vivax* gametocytes is further complicated by the lack of stable culture methods, and also because clinically they appear early in the infection – transmission may have happened before a patient seeks medical attention.

(c) **Hypnozoite treatment and relapse prevention.** For *P. vivax* and *P. ovale*, an additional hurdle is to prevent relapse of the hypnozoite or dormant liver form. Attempts are underway to develop biological assays for efficacy, and first successes have been achieved [163]. The availability of an assay means that it is now possible to profile new chemical series to see whether they have activity against both the blood and the liver stages. This has led to some success in hits-to-leads chemistry, which shows activity against hypnozoites.

(d) **Chemoprevention.** The most advanced vaccine against malaria is the RTS,S vaccine (currently planned for launch in 2015 for children, which caused a 50% reduction in incidence of acute falciparum infections in young children in initial Phase III studies [164-166]). There remains a significant need for a molecule which can protect the adult population from infection. As malaria becomes less common in Africa, the natural immunity within the population will fall, and the continent’s population would be as at risk of malaria infection as western travellers are today. New protective medicines will need long half-lives. Depot formulations are possible, but then the medicines must be potent with a human daily dose of less than 10 mg, and preferably less than 1 mg.

Not all molecules meet all criteria above, as new malaria medicines will be a combination of several active ingredients. Understanding the activity of the compounds against each stage in the *Plasmodium* life cycle (a Malaria Lifecycle Fingerprint) is important as early as possible in the development process to allow a focused development strategy and prioritization between molecules.

In addition, three factors are critical for the development of new medicines. First, safety and tolerability; since these medicines will be used in areas where pharmacovigilance systems are not always completely effective. Second, an appropriate speed of action; for blood stage medicines, the patients have become used to the speed of action of artemisinin. Third, medicines need to be potent; many of the current medicines are active at adult doses approaching 1 g per day. If the next-generation of medicines are more potent, this can greatly impact the cost of treatment. Cost reductions will be much more likely to come from doses that are lower than current therapy, rather than from simpler, cheaper molecules.

## Conclusions

The pipeline of new molecules targeting malaria is much richer now than it was two years ago [167]. Two significant events have occurred: first, new medicines have moved down the pipeline, specifically the prequalification of artesunate for severe malaria and the stringent regulatory approval of DHA-piperaquine and pyronaridine-artesunate as new fixed-dose anti-malarials. Second, and arguably more interesting, is the number of new molecules and new classes of molecules that are entering the pipeline. There are at least seven new compound families that have been discovered in the past five years. This is a rich portfolio and reflects the commitment of the field as a whole. Nonetheless, from a portfolio point of view there are still gaps. The chance that a new molecule entering Phase I studies will make it to registration is still around 20% for anti-infectives, and for a new combination two molecules would be needed. In addition, new TPPs have emerged from the malaria eradication agenda and compounds need to be measured against these, focusing on transmission-blocking and anti-relapse potential. With the sustained commitment of all of partners – donors, scientists, academics and the pharmaceutical industry – the next decade will be a very exciting time to be in clinical development of new therapeutics for malaria.

## Abbreviations

ACPR28: Adequate clinical and parasitological response at day 28 post treatment; ACT: Artemisinin combination therapy; AMFm: Affordable medicines facility – malaria; Sino FDA: Chinese authorities; DHA: Dihydroartemisinin; DNDi: Drugs for Neglected Diseases initiative; EMA: European Medicines Agency; FDA: Food & Drugs Administration; G6PD: Glucose-6-phosphate dehydrogenase; IPTp: Intermittent preventive treatment in pregnancy; MMV: Medicines for Malaria Venture; SLS: Sublingual sugar; TPPs: Target Product Profiles; TDR: UNICEF-UNDP-World Bank-WHO Special Programme for research and training in Tropical Diseases; WHO: World Health Organization.

## Competing interests

The authors declare that they have no competing interests, beyond the fact that MMV is involved in supporting the development of some of these medicines. This paper was designed as an objective review of the entire worldwide portfolio of medicines in development, and the authors apologise in advance to all colleagues where space constraints prevented a fuller description of the molecules.

## Authors’ contributions

MA and TW prepared the data tables; the commentary was written by TW, JB, JM and SD. All authors read and approved the final manuscript.
